# Mechanical and Corrosion Properties of Laser Surface-Treated Ti13Nb13Zr Alloy with MWCNTs Coatings

**DOI:** 10.3390/ma13183991

**Published:** 2020-09-09

**Authors:** Beata Majkowska-Marzec, Patryk Tęczar, Michał Bartmański, Bartosz Bartosewicz, Bartłomiej J. Jankiewicz

**Affiliations:** 1 Department of Materials Engineering and Bonding, Faculty of Mechanical Engineering, Gdansk University of Technology, G. Narutowicza 11/22, 80-233 Gdansk, Poland; patryk.teczar@gmail.com (P.T.); michal.bartmanski@pg.edu.pl (M.B.); 2 Institute of Optoelectronics, Military University of Technology, gen. S. Kaliskiego 2, 00-908 Warsaw, Poland; bartosz.bartosewicz@wat.edu.pl (B.B.); bartlomiej.jankiewicz@wat.edu.pl (B.J.J.)

**Keywords:** Ti13Nb13Zr alloy, laser treatment nanoindentation, electrophoretic deposition, carbon nanotubes, potentiodynamic polarization

## Abstract

Titanium and its alloys is the main group of materials used in prosthetics and implantology. Despite their popularity and many advantages associated with their biocompatibility, these materials have a few significant disadvantages. These include low biologic activity—which reduces the growth of fibrous tissue and allows loosening of the prosthesis—the possibility of metallosis and related inflammation or other allergic reactions, as well as abrasion of the material during operation. Searching for the best combinations of material properties for implants in today′s world is not only associated with research on new alloys, but primarily with the modification of their surface layers. The proposed laser modification of the Ti13Nb13Zr alloy with a carbon nanotube coating is aimed at eliminating most of the problems mentioned above. The carbon coating was carried out by electrophoretic deposition (EPD) onto ground and etched substrates. This form of carbon was used due to the confirmed biocompatibility with the human body and the ability to create titanium carbides after laser treatment. The EPD-deposited carbon nanotube coating was subjected to laser treatment. Due to high power densities applied to the material during laser treatment, non-equilibrium structures were observed while improving mechanical and anti-corrosive properties. An electrophoretically deposited coating of carbon nanotubes further improved the effects of laser processing through greater strengthening, hardness or Young′s modulus similar to that required, as well as led to an increase in corrosion resistance. The advantage of the presented laser modification of the Ti13Nb13Zr alloy with a carbon coating is the lack of surface cracks, which are difficult to eliminate with traditional laser treatment of Ti alloys. All samples tested showed contact angles between 46° and 82° and thus, based on the literature reports, they have hydrophilic surfaces suitable for cell adhesion.

## 1. Introduction

In recent decades, a significant increase in the number of diseases of the osteoarticular system has been observed—especially the knee and hip joints. These diseases are an increasingly common cause of joint destruction, which often requires replacing the joints with artificial ones. This problem has increased the demand for implants and prostheses. Related research and modifications—as well as combinations of engineering materials used and their surfaces—constitute wide scientific interest [1].

Titanium and its alloys is one of the most widely used groups of materials for the production of orthopedic implants and prostheses. Due to the properties of titanium, titanium-based materials cover almost 40% of the current biomaterial market. This is particularly related to their mechanical and functional properties being similar to hard bone tissues (especially Young′s modulus), good biocompatibility and better corrosion resistance than austenitic steels and many other bioalloys. However, the restrictions on their growth with fibrous tissue, the possibility of inflammation and allergic reactions, and poor wear resistance are still a drawback in their use [1,2,3].

The titanium alloys Ti6Al4V, Ti6Al7Nb and Ti13Nb13Zr are often used in medical applications. Alloys with the addition of zirconium and niobium eliminate the adverse effects of aluminum and vanadium on the nervous system, the possibility of metallosis and the initiation of diseases (including cancers or Alzheimer′s disease). In addition, they have better corrosion resistance, and a Young′s modulus value similar to longitudinal bone tissue. However, they still have insufficient abrasive resistance [3,4].

Due to the demand for more appropriate properties of titanium materials, many modification methods have been developed. Some of these are associated with the modification of surface topography, mechanical and physicochemical or biologic properties, resulting in increased adhesion. The simplest modification methods include chemical etching to increase roughness and osseointegration [2,5,6]. Among the many modification techniques used there are electrochemical oxidation which increases adhesion [7] or polishing, which has a significant impact on increasing the anti-corrosive properties [8]. Improvement of corrosion properties and reduction of bacterial adhesion have been achieved in the hydrophilic synthesis process [9]. These materials have also been subjected to anodizing processes [10] and processed in alkaline solutions, which resulted in high bioactivity of the Ti13Nb13Zr alloy [11]. In addition, titanium alloys have been oxidized in an acid solution in the presence of fluoride ions to obtain a nanoparticle oxide layer with good adhesion, high mechanical properties and increased bioactivity [12]. The improvement of titanium biomaterials properties has been obtained by subjecting them to ion implantation [13,14], plating [15,16] and nitriding [17,18]. A popular method of obtaining better properties of the titanium alloys tested is coating them using silicates [19], chitosan [20,21], or phosphates including hydroxyapatite (HAp) [22,23,24,25] and nanoHAp [7,26,27,28]—together with their composite combinations with other elements [26,28,29,30,31,32,33]—thus facilitating better adhesion and antibacterial properties. Carbon and diamond-like coatings [34,35,36,37,38,39] and their composite combinations with other elements [40,41,42], as well as carbon nanotubes (CNTs) [41,43,44,45] and their composite combinations with other elements [31,45,46] have been tested to obtain better mechanical (especially nanohardness) and anti-corrosive properties. The evolution of multi-walled carbon nanotubes (MWCNTs) for in situ TiC formation during spark-plasma sintering in titanium metal matrix composites allows significantly enhancing the mechanical and tribological properties of the composites [47]. The densification and microhardness of the sintered nanocomposites with MWCNTs additions have been shown improve tremendously with an increase in sintering temperatures [48]. Positive effects in the form of high value of material hardness and excellent wear resistance result from the laser-cladding of MWCNTs on Ti, leading to TiC-reinforced Ti-matrix composite layers [49]. Additionally, the layer of carbon nanotubes (MWCNTs) deposited on titanium via the electrophoretic (EPD) method provides better compatibility of the implant with the body tissues. Carbon coatings allow choosing the best time for the protein to form a stable connection with the surface of the titanium implant [50]. Modern laser technologies have been used in the modification of biocompatible materials such as titanium. These modification methods allow melting and remelting [51,52,53], alloying [17,44,54], direct laser deposition [55], texturing and marking [56,57] and laser ablation [58]. Interest in laser methods is increasing due to their numerous advantages including the possibility of local only processing. Laser modifications are characterized by high process efficiency, lower cost of industrial use and possibilities of process automation in industrial production, no deformation of the workpiece and great flexibility in modification of the processed material structure. The concentration of high-power densities of materials subjected to laser modification for just a few milliseconds allows observing structures different from equilibrium for this material. These results, among others, consist of improvement of mechanical properties, increase in their hardness and often improvement of their anti-corrosion properties. However, these positive changes are often accompanied by deterioration in surface quality, which is one of the disadvantages of laser surface modification techniques of most materials [17,23,33,44,52,53,54,56,57,59,60,61,62].

This study aims to assess the impact of laser modification using an neodymium-doped yttrium aluminum garnet laser (Nd:YAG) ((TruLaser Station 5004, TRUMPF, Ditzingen, Germany) with appropriately selected parameters on the microstructure, mechanical, physical and corrosion properties of the surface layer of the Ti13Nb13Zr titanium alloy with an electrophoretic deposited layer of multi-walled carbon nanotubes. The selected process parameters are the result of many melting and laser alloying processes carried out at the testing stage. The proposed laser modification of the Ti13Nb13Zr surface with a carbon nanotubes coating allows to obtain better mechanical properties and a hydrophilic surface.

## 2. Materials and Methods

### 2.1. Preparation of Materials

#### 2.1.1. Preparation of Titanium Samples

A Ti13Nb13Zr alloy rod provided by a commercial supplier (Xi’an SAITE Metal Materials Development Co., Ltd., Xi’an, China). with chemical composition detailed in Table 1 was used. A rod (with a diameter of 40 mm) was cut using a precision cutter (Brillant 220, ATM GmbH, Mammelzen, Germany) into 4-mm-thick slices and divided into quarters. Each of the quarters was cut at the edge to fix the copper wire needed at a later stage of the work—the EPD process. Abrasive machining was carried out on a metallographic grinding machine (Saphir 330, ATM GmbH, Mammelzen, Germany) by the wet method to remove impurities and deformation as well as level the surface. Samples were sanded with SiC sandpaper with 220, 500 and 800 gradations. Along with the transition between individual sandpaper gradations, the samples were thoroughly washed and dried. The surface roughness of the substrates was adjusted to values within Ra 0.24 ± 0.08 µm.

**Figure 1:**
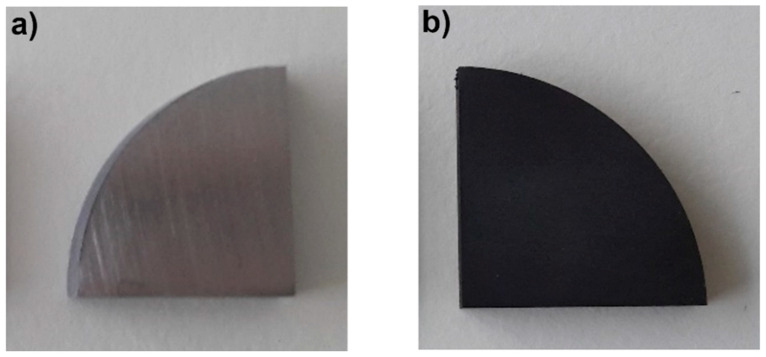
Alloy sample surfaces after (**a**) abrasive treatment and (**b**) etching in 5% hydrofluoric acid (HF).

The samples were washed with acetone (Chempur, Piekary Śląskie, Poland) and distilled water and etched for 20 s in 5% hydrofluoric acid (HF) (Chempur, Piekary Śląskie, Poland), then washed again with distilled water and dried. Etching was performed to thoroughly clean them from impurities and develop the surface of the prepared samples. The surface image of the Ti13Nb13Zr alloy samples after abrasive machining and etching is shown in Figure 1.

#### 2.1.2. Preparation of Suspension of Functionalized Carbon Nanotubes

The electrophoretic suspension was prepared from distilled water and multi-wall carbon nanotubes MWCNTs (3D Nano, PlasmaChem GmbH, Berlin, Germany) in loose form with an external diameter of 5–20 nm, number of walls of 3–15, internal diameter of 2–6 nm and length of 1–10 µm.

Before preparing the suspension, the carbon nanotubes were functionalized using a method described elsewhere [46]. The chemical modification allowed for the introduction of carboxyl groups on the surface of the nanotubes. These groups provide a negative charge for nanotubes and allow their electrophoretic seating on the anode. Functionalization of CNTs makes the process of their deposition by EPD faster and easier to occur. This process also affects the morphology of nanotubes, i.e., their length decreases [63], which results in better adhesion to the surface.

At first, 480 mg MWCNTs were annealed in a vacuum furnace (20VP-411/14 hV, Seco/Warwick SA, Świebodzin, Poland) at 400 °C for 8 h. Then, the nanotubes were dispersed by means of ultrasound in an ultrasonic homogenizer (Bandelin Sonopuls HD 2070, Berlin, Germany) in a small amount of distilled water and added to 200 mL of a 3:1 *v*/*v* mixture of H_2_SO_4_ and HNO_3_ (Chempur, Piekary Śląskie, Poland). The resulting suspension was heated for 2 h at 70 °C on a heating plate. Then, after cooling to room temperature, the suspension was centrifuged (MPW-251, MPW MED. INSTRUMENTS, Warszawa, Poland) and washed several times with water until a neutral pH was reached. Finally, a suspension of MWCNTs in distilled water with a concentration of 0.19 wt% was obtained.

### 2.2. Electrophoretic Deposition of Carbon Nanotube Coating

The electrophoretic deposition (EPD) was carried out in a 0.19% suspension of MWCNTs in distilled water. Ti13Nb13Zr alloy samples constituted anodes in this process. The opposite electrode—the cathode—was platinum in this system. The electrodes were placed parallel to each other at a distance of about 10 mm and then connected to a DC power supply (MCP/SPN110-01C, MCP Corp., Shanghai, China). EPD was carried out at 20 V for 30 s at room temperature. After the deposition process, the samples were dried at 80 °C for 40 min in a laboratory dryer (SLN32, POL-EKO Aparatura, Wodzisław Śląski, Poland).

### 2.3. Laser Modification

The samples (T–Ti13Nb13Zr alloy after grinding and C–Ti13Nb13Zr alloy with MWCNTs coating) were subjected to laser treatment in order to develop the surface. This treatment was performed using a Nd:YAG pulse laser (TruLaser Station 5004, TRUMPF, Ditzingen, Germany) with the parameters described in Table 2. The term laser-melted corresponds in our manuscript to laser processing of titanium alloys, where the properties are changed without changing the chemical composition of the surface layer, while term laser-alloyed is used for laser processing of Ti13Nb13Zr alloy with a previously deposited nanotube coating leading to laser carburizing.

This process was carried out under a protective gas with an argon (Ar) content of not less than 99.987% for the proper course of remelting the substrate.

### 2.4. Surface and Cross-Sections Evaluation of Samples

#### 2.4.1. Preparation of Samples for Surface and Cross-Section Tests

The samples T(1 ÷ 6), C(1 ÷ 6) and native material—MR—were integrated in phenolic resin (Verte 602, Lamplan, Gaillard, France) in a laboratory press (Opal 410, ATM GmbH, Mammelzen, Germany) and subjected to grinding on a metallographic grinding machine (Saphir 330, ATM GmbH, Mammelzen, Germany) using sandpapers with a gradation of 180, 400, 600, 800, 1200, 2000 and 4000 and polishing using diamond paste (DP-Paste M 1 μm, Struers, Inc., Cleveland, OH, USA). The samples were then etched in Kroll′s reagent (a mixture of HNO_3_ and HF acids in distilled water in a 6/2/92 volume ratio).

#### 2.4.2. Analysis of Surface Phase Composition

The samples MR, C, T(2,5), C(2,5) were subjected to phase structure tests using an X-ray diffractometer (X′Pert Pro, Philips, Amsterdam, The Netherlands) with a goniometer PW 3050/60 (θ–θ) with a Cu copper lamp (Kα1 = 1, 5405980 Å, Kα2 = 1.5444260 Å, Kα2/Kα1 ratio = 0.5). The test (with an 2θ angle range of 20–60° at 40 V, 30 mA and scanning speed 0.02/1.5 °/s) permits the determination of the crystal structure of the material.

#### 2.4.3. Raman Spectroscopy

Raman measurements were carried out using Renishaw InVia Raman microscope (Renishaw plc., Wotton-under-Edge, UK) equipped with an EMCCD detector (Andor Technology Ltd, Oxford Instruments, Belfast, UK). The Raman signal was acquired using laser radiation with a wavelength of 532 nm (27 mW). The laser beam was directed to the sample through a 50× objective lens. The wavelength of the instrument was calibrated using an internal silicon wafer, and the spectrum was centered at 520.5 cm^−1^. Raman spectra measurements were made for maps consisting of 100 points per sample. Based on this, an average spectrum was obtained and the standard deviation of the signal was determined—which is an indicator of the homogeneity of the layers.

#### 2.4.4. Surface and Cross-Section Morphology and Topography and Chemical Analysis

The microstructure of the surface before and after corrosion tests and cross-sections of samples were observed using an optical microscope (UC50, Olympus Europa SE & Co. KG, Hamburg, Germany) and a scanning electron microscope SEM (JSM-7800F, JEOL, Tokyo, Japan). Microscopic images of specimens using the SEM were taken at an accelerating voltage of 1 kV. The thickness of the MWCNTs coating deposited on the Ti13Nb13Zr alloy was measured on the cross-section and after its local detachment using the adhesive force from the edge of the sample. Elemental composition analysis on the cross-section of the laser-modified samples was taken using an X-ray energy dispersion spectrometer (EDS) (Octane Elite 25, EDAX Ametek, Berwyn, PA, USA).

#### 2.4.5. Surface Wettability

The water contact angle measurements—before and after laser processing of the Ti13Nb13Zr alloy with and without carbon coating—were made using an angular contact goniometer with a computer set (Contact angle goniometer, Zeiss, Ulm, Germany). Measurements were made by the falling drop method. The results comprise the arithmetic means of three measurements carried out at room temperature after 10 s.

#### 2.4.6. Surface and Cross-Section Nanomechanical Properties

Nanoindentation tests were carried out on the surface and on cross-sections for MR, T(2,4) and C(2,4) using a nanoindenter equipped with a pyramidal trihedral diamond Berkovich indenter with an apical angle of 124.4° (NanoTest Vantage, Micro Materials, Wrexham, UK). The measurements were made with a maximum force of 50 mN. The rise time from zero value was 10 s, and the stop time with the maximum force was 5 s. The samples were unloaded within 4 s. A total of 115 measurements (5 × 13) were made every 30 μm for each sample on the cross-section and 115 measurements (A × B) every 30 μm on surfaces.

Hysteresis curves of load–strain were recorded during the study. The reduced Young′s modulus (E_IT_) and the nanohardness (H_IT_) were determined based on the Olivier–Pharr method [64,65] using the NanoTest results analysis program. Calculations of the real Young′s modulus (Es) were made using method developed by Olivier and Pharr [66].

### 2.5. Corrosion Tests

#### 2.5.1. Preparation of Samples for Corrosion Tests

Copper wires were attached to uncoated and MWCNTs-coated Ti13Nb13Zr samples unmodified and modified using laser radiation. The back and side surfaces of the samples and their connection to the wire were isolated with a two-component epoxy ceramic coating (BELZONA 1321, Belzona, Inc., Harrogate, UK) to protect against corrosion. The coating was made of 4 parts base and 1 part of hardener. The partially isolated samples were allowed to dry for 48 h.

#### 2.5.2. Corrosion Measurements Using the Potentiodynamic Polarization Method

Corrosion tests were carried out for samples MR, C, T2, T4, C2 and C4 in a three-electrode configuration: reference electrode (RE)—calomel electrode, auxiliary electrode (CE)—platinum electrode, working electrode (WE)—tested sample. Then, 250 mL of Ringer′s solution, heated to 37 °C by a heating plate (MS-H-PRO +, Dragon Laboratory Instruments, Ltd., Beijing, China) was used as the electrolyte during the corrosion process.

The tests were carried out using a potentiostat (ATLAS 0531, ATLAS-SOLLICH, Rębiechowo Poland) equipped with AtlasCorr05 control and cooperating software (ATLAS 0531, ATLAS-SOLLICH, Rębiechowo, Poland). The corrosion tests began by determining the stationary potential as a function of time to determine the corrosion tendency of the material. After the potential values stabilized, potentiodynamic studies were started. The potential increased at a speed of 1 mV/s. A potential range 1–2 V was used. Individual polarization ranges were selected based on the stationary potential.

## 3. Results and Discussion

### 3.1. Measurement of MWCNTs Coating Thickness

**Figure 2:**
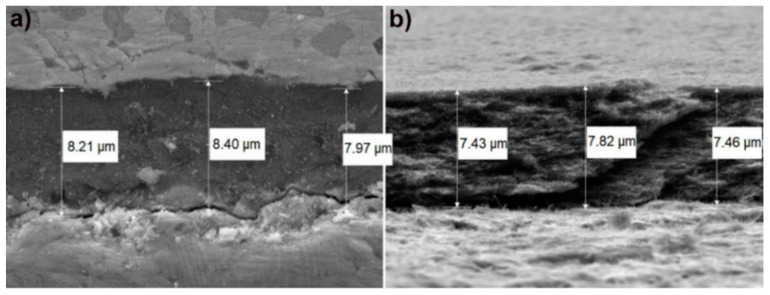
SEM images of Ti13Nb13Zr alloy MWCNTs coatings. (**a**) Cross-section; (**b**) fracture, with indicated measured thicknesses at three different points.

**Figure 3:**
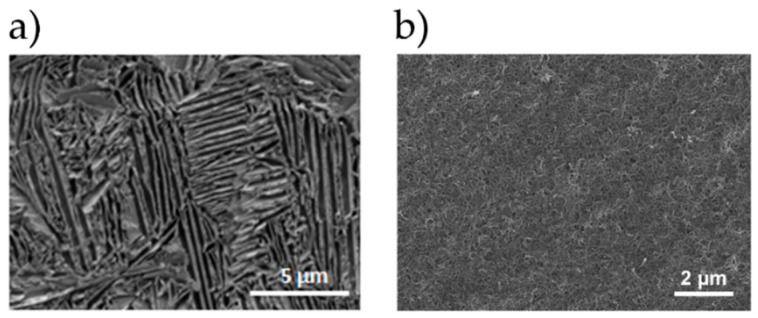
SEM images of Ti13Nb13Zr alloy before laser treatment. (**a**) MR; (**b**) MR with MWCNTs coatings.

The thickness of the electrophoretically deposited MWCNTs coatings was measured by using SEM. The measurement was made on the cross-section of the sample (Figure 2a) and its fracture (Figure 2b). The presence of uniformly deposited carbon nanotubes on the Ti13Nb13Zr substrate is shown in Figure 3b. The critical determinant of any coating is its adhesion to the substrate. Such measurements have been already performed for this type of coatings and published in [46], demonstrating this property suitable for coatings on titanium implants. The average thickness of MWCNTs coating was 7.88 μm ± standard deviation of 0.35 μm.

### 3.2. Morphology and Topography Studies

**Figure 4:**
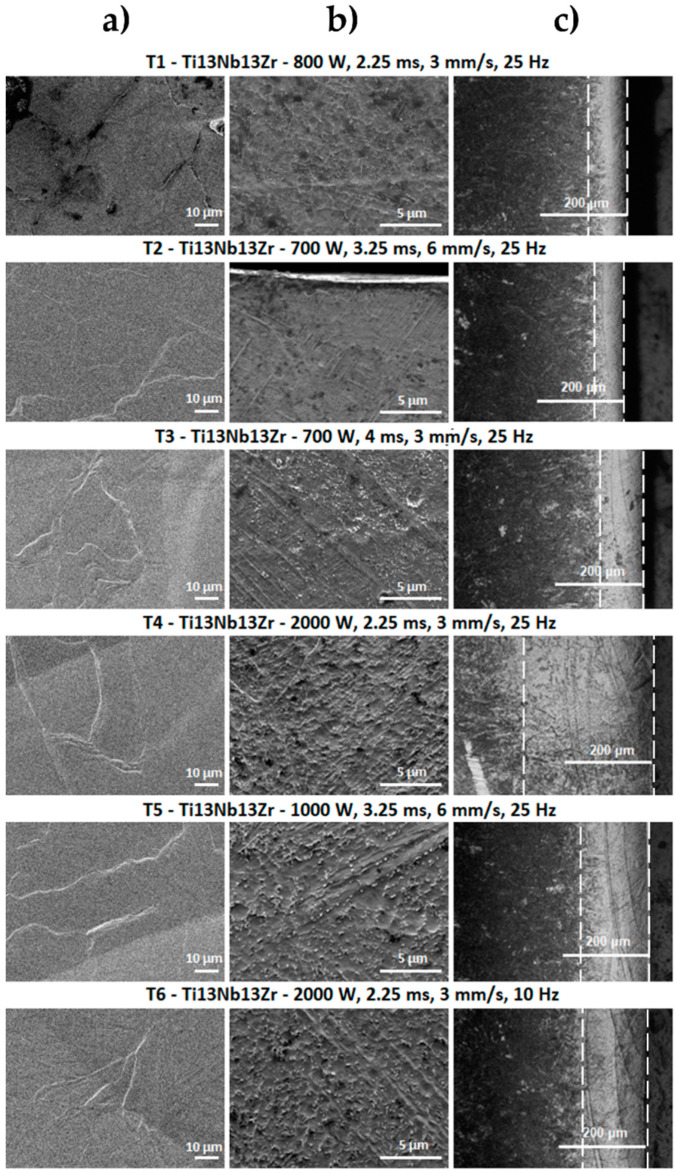
SEM images of Ti13Nb13Zr alloy after laser processing. (**a**) Surface topography; (**b**) cross-sections; (**c**) images from an optical microscope.

**Figure 5:**
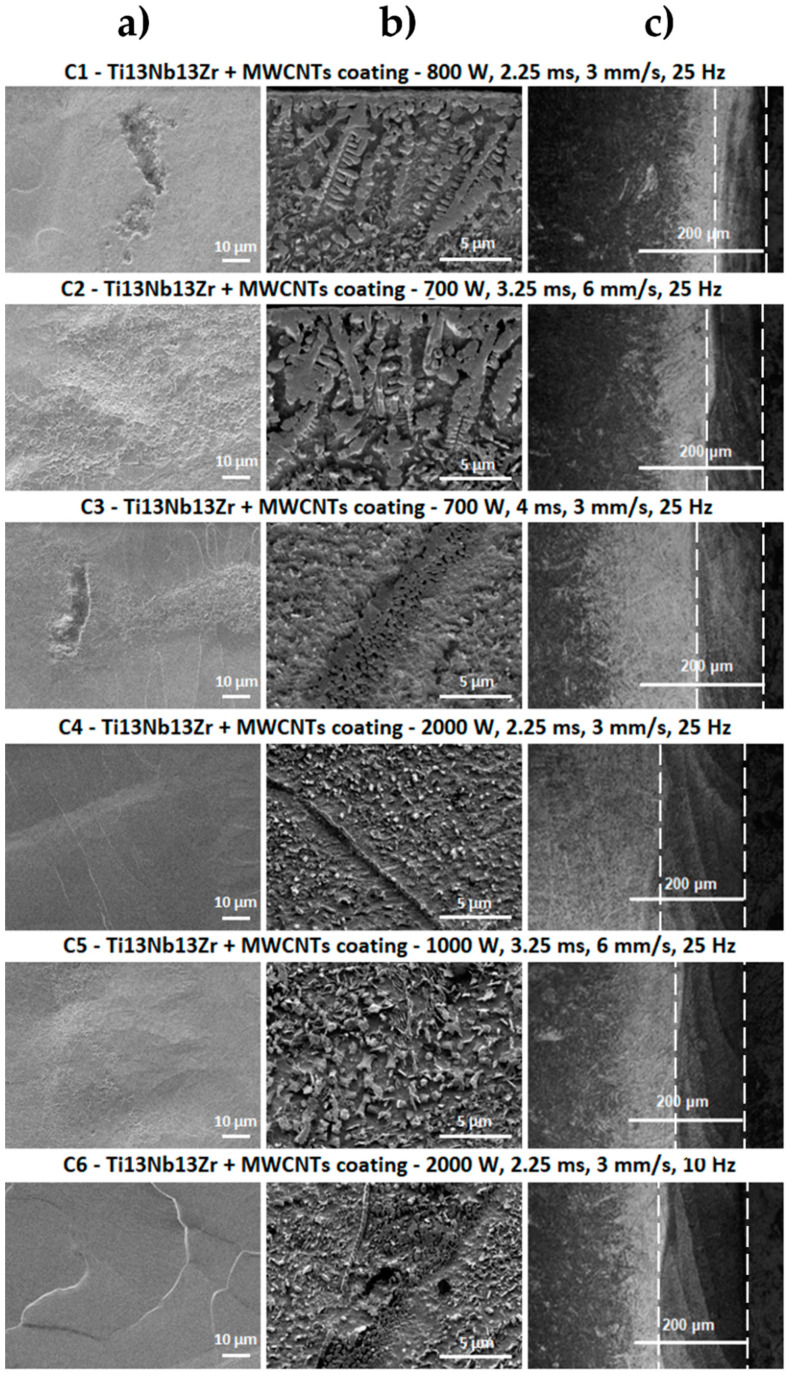
SEM images of Ti13Nb13Zr alloy with MWCNTs coating after laser processing. (**a**) Surface topography; (**b**) cross-sections; (**c**) images from an optical microscope.

The optical microscope and scanning electron microscope images of the surface and cross-sections of the tested samples of Ti13Nb13Zr alloy before and after laser treatment with and without a coating of carbon nanotubes are shown in Figure 3, Figure 4 and Figure 5.

Based on the analysis of the surface morphology of the laser-processed samples (Figure 4), it can be seen that the T1 sample did not have the characteristic changes normally attributed to the laser processing processes, but only changes that can indicate a strong overheating of the material as in [52]. The small remains from remelting—which may constitute remelted material splinters—are visible on its surface. The remaining laser-melted layers had scratches and bumps characteristic for laser processes, which were the remaining effects of the laser beam pulse. This was especially visible for laser processed samples with higher laser pulse power (T4 ÷ T6). The laser beam penetrated deeper into the material shown on the image of cross-sections from the optical microscope in Figure 4, which caused greater mixing of the material and solidification in a more layered manner. No cracks were observed on the surface of the samples.

The SEM image of laser-processed surfaces with a previously deposited coating of multi-walled carbon nanotubes (Figure 5) reveals major changes occurring on the surface of the material. For samples treated with lower laser pulse powers—in which the laser beam penetrates the shallower layer of the substrate (shown in cross-sectional images from the optical microscope)—clusters remaining from the MWCNTs coating are visible. This indicates that the laser modification did not melt the entire area. In places where the laser-melted area is discernible, changes in structure were observed, which are represented by numerous folds, bulges and secretions. For samples where the laser beam with higher power penetrated the material deeper (T4 ÷ T6), these changes were less visible, which may have been associated with greater penetration of the carbon coating into the Ti13Nb13Zr alloy. Surface unevenness for these samples was greater than for laser-melted samples without coatings. No cracks were observed.

The SEM image of the Ti13Nb13Zr alloy (Figure 3a) is characteristic for this material after chemical etching. The presence of one phase tiles in the matrix of the second phase can be observed. SEM images for laser-treated samples in the laser-melted layer directly at the sample front were taken (Figure 4b). For laser-modified samples with 700- and 800-W power, changes in the structure and grain refinement are seen. A typical martensitic transformation is noted. The microstructure of the T1 ÷ T3 samples was very similar, with the changes in the microstructure reaching the deeper material layers for the T3 sample. The microstructure of laser-melted layers made with greater power and laser pulse (T4 ÷ T6) indicates a much stronger mixing of components that are in a liquid state before crystallization in the laser beam pool. No visible MR phase plates were observed. This indicates a strong grain refinement. Despite the visible boundaries of the laser beam paths, no microstructure changes were observed in these places. Visible boundaries between the laser-fused layer, the layer below the fusion (which was affected by the heat of the laser beam) and the intact material were observed. We observed the deepest laser beam penetration in samples T4 and T6 visible as a bright band from the surface (Figure 4c).

**Figure 6:**
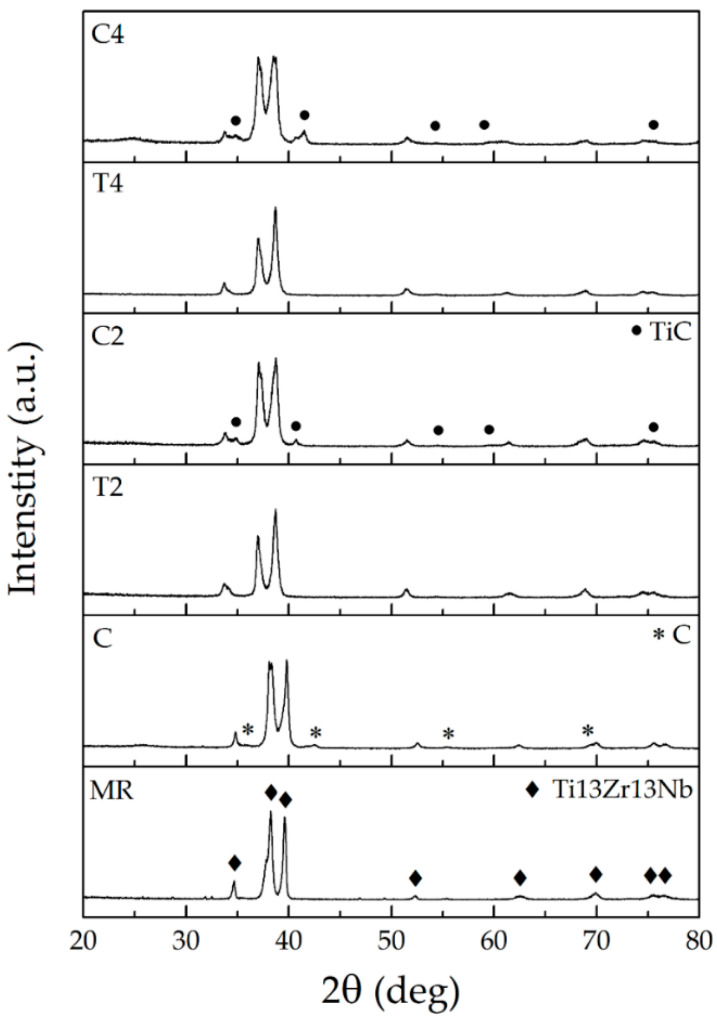
XRD spectra of Ti13Nb13Zr alloy for: MR, C (MR with MWCNTs coating), T2, T4—its alloys after laser treatment, C2, C4—its alloys with MWCNTs coating after laser treatment.

As can be seen in the SEM images in Figure 4 and Figure 5, laser-alloyed samples differed significantly in microstructure from laser-melted samples. This is the result of introducing a new element in the structure of the substrate (Ti13Nb13Zr alloy), which is carbon derived from the MWCNTs coating. Knowing about the results of XRD (Figure 6 and the Ti–C phase equilibrium plot, the newly formed phases are identified with TiC titanium carbide. They are formed due to the liquid state of the substrate and the coating applied to it under the influence of a high temperature laser beam of high power. Mixing of the two components and their crystallization results in the formation of grains characteristic for the surface zone after laser processing and structures resulting from the enrichment of the substrate with the coating element [67,68]. These structures are observed in the SEM images (Figure 4b and Figure 5b) of each sample and they take different forms. Their presence is most densely observed in the shallowest laser-melted layers, where the coating material was melted into a smaller volume of the substrate material. In the C1 and C2 section (Figure 5b), TiC dendrites are observed at the entire penetration depth. TiC structures in the C3 microstructure turning into a structure consisting of spheres for which the pulse duration was almost twice as large. Based on [67], it is noted that a symmetrical dendritic structure in the form of titanium and carbon cubic subnets tends to grow into a spherical form to reduce interfacial energy. It is concluded that increasing the duration of the laser beam pulse and reducing its feed speed reduce the solidification rate of the material and smooth the convective flows in the alloy material. This promotes the formation of TiC in a spherical form which is also favored by the increase in laser pulse power. This is observed in the images for samples C4 ÷ C6 (Figure 5b), where the laser-melted layer is clearly deeper, and the substrate material was melted into the larger volume of the substrate material. The quantity of TiC phases is more numerous for areas of the laser-melted layer closer to the sample front. The smallest forms of TiC phases are observed in samples C4 and C6. In the SEM images of cross-sections one can see the boundaries of individual laser beam transitions (clear lines in samples C4 and C6 in Figure 5b). In these places, more spheres with smaller diameters were observed. This may have been the result of re-remelting the material during the next laser beam transition. For this method of machining, the Ti13Nb13Zr alloy combined with the enrichment of this material in carbon, the presence of cracks in the modified layer is not observed. This is an advantage of the presented modification, because traditional laser-processing of titanium alloys is most often associated with formation of surface cracks, which are difficult to eliminate.

The number of TiC structures in the form of dendrites was much smaller in the present study compared to [67,68]. This is probably due to the thickness of the carbon coating applied on the titanium alloy. In the cited studies [67,68], the coating was over 10-fold and 30-fold thicker than the EPD obtained in this process. The amount of carbonaceous material fused into a similar volume of substrate material was, therefore, clearly larger. The coating itself was made of a different carbon material and applied in a different manner. Carbon nanotubes were selected as a carburizing additive because of their mechanical and chemical stability and their tubular shape translating into increased mechanical properties of the laser-modified surface layers. In addition, the EPD process ensured obtaining repeatable coatings with a predefined thickness. It was also important that they are considered to be biocompatible materials with the human tissue.

The analysis of the cross-sections observed on the optical microscope allowed the assessment of the penetration depth and layers of material that could be affected by laser processing. Dependence of material depth on the parameters used was observed. As the laser pulse power increased at a constant feed rate and frequency, more than a double deepening of the laser-melted layers was observed between T2 and T5. A fourfold increase in the depth of the modified layer was seen between T1 and T4, where a power increase was applied. No significant macroscopic changes were seen with a slight decrease in the pulse power between T1 and T2, a simultaneous increase of feed speed and extension of the pulse duration. By maintaining the same power between T2 and T3 and increasing the pulse duration and reducing the feed speed, a significant penetration deepening was seen. Between T4 and T6, a frequency reduction was used with other fixed parameters. A shallowing of the laser-melted layer was observed. Optical microscope images for laser carburized samples revealed laser-melted layers with a characteristic, darker color. This was the result of carburizing the material. Similar relationships were observed as for material modification without a carbon coating. Based on this analysis, it was decided that the next tests would be carried out on samples with the shallowest and deepest laser-melted layers: T2, T4, C2 and C4. Another aspect in their selection was the different form of TiC in the C2 and C4 samples.

### 3.3. Chemical and Phase Analysis, Raman Spectroscopy

**Figure 7:**
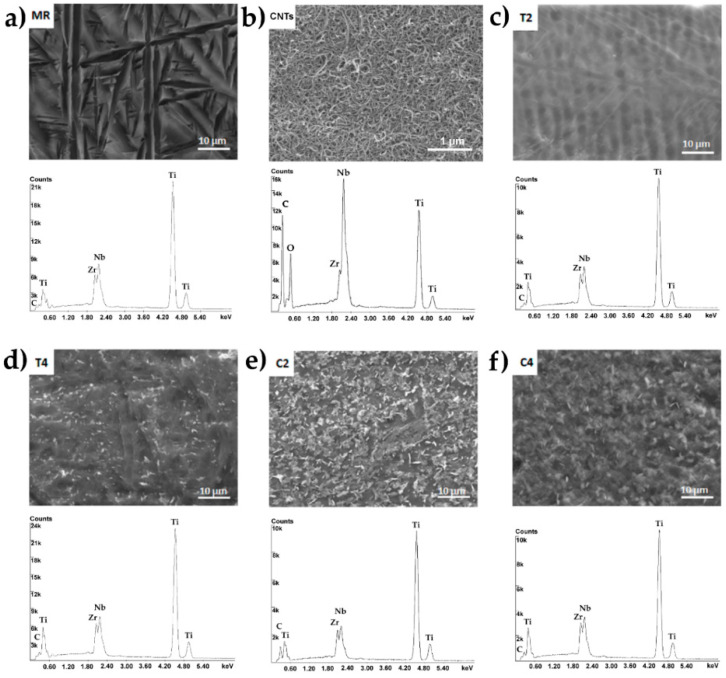
EDS spectra of Ti13Nb13Zr alloy: (**a**) MR, (**b**) MR with MWCNTs coating, (**c**,**d**) T2, T4—its alloys after laser treatment, (**e**,**f**) C2, C4—its alloys with MWCNTs coating after laser treatment with the shallowest penetration.

**Figure 8:**
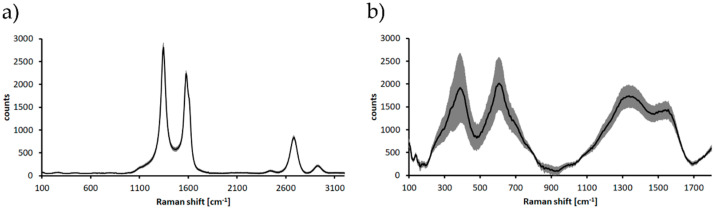
Raman spectra of (**a**) MR with MWCNTs coating (black line—average Raman spectrum; gray area–standard deviation of the signal); (**b**) TiC layer recorded for sample C2 (black line—average Raman spectrum; gray area—standard deviation of the signal).

The X-ray energy dispersion spectroscopy (EDS) spectra for the reference sample Ti13Zr13Nb and laser processed and carburized samples with the smallest and largest penetration depth obtained (T2, T4, C2, C4) are shown in Figure 5. The tests were carried out at a distance of 10 μm from the sample surface, for cross-sectional areas of 50 × 80 μm. Three main alloying elements were observed in the EDS spectrum of the reference sample: titanium (the most intense peak), zirconium and niobium. The spectra for laser melted samples (T2 and T4) did not differ much from the result for the reference sample. A quantitative analysis showed a smaller content of the alloying element Zr, which could indicate that this component was more chemically etched after laser processing. The smaller content of this alloying element can also result from temporary chemical composition fluctuation during laser melting and an appearance of lighter elements on the surface. For laser carburized samples (C2 and C4)—and especially for the sample with the shallowest penetration depth (C2)—a more pronounced peak for carbon in the EDS spectrum and an increase in the quantitative content of this element in the material were noted. The more intense carbon peak in EDS spectra, especially for sample C2 (Figure 7) and presence of dendritic and spherical TiC well visible in microstructure of surface zone (Figure 5b), confirmed additionally by XRD (Figure 6) and Raman (Figure 8) analysis, prove effect of laser carburization.

The X-ray diffraction patterns obtained for a reference material sample (Ti13Nb13Zr) and with MWNCNTs coating as well as samples after melting and alloying with the shallowest and deepest penetration (T2, T4, C2, C4) are shown in Figure 6.

The highest peaks observed on the diffractograms are indexed as derived from the substrate material-Ti13Nb13Zr alloy. After laser processing, this material presented spectra similar to a laser-unprocessed material. For an alloy with a coating of carbon nanotubes, the presence of carbon-specific peaks (marked with an asterisk in Figure 6) is observed in addition to the peak characteristic of the substrate material. Samples after laser carbon alloying in their spectra revealed peaks suitable for the substrate alloy and characteristic for the new phase—titanium carbide (marked with a dot symbol in Figure 6). Such spectra were obtained previously in [67,68], where graphite was used as carburizing material.

The main mechanism of TiC formation in laser processing involves the diffusion of C atoms into liquid Ti, followed by nucleation and growth of TiC crystals. It is possible for titanium carbides to be formed in a range of about 10–19% by carbon weight in the temperature range 1648–3000 °C Naccording to the Ti–C phase equilibrium system [69,70].

In the Raman spectrum of the MWCNTs coating (Figure 8a) a typical spectral pattern was observed. The spectrum is characterized by the occurrence of the following bands: 1345 cm^−1^ (D band), 1580 cm^−1^ (G band) and 2690 cm^−1^ (2D band) [71]. In the Raman spectrum of sample after laser modification (Figure 8b) the characteristic spectral bands for titanium carbide were observed at 400 cm^−1^, 610 cm^−1^, 1340 cm^−1^ and 1560 cm^−1^ [72].

### 3.4. Wettability Analysis

The values of the average contact angle for uncoated and MWCNTs-coated Ti13Nb13Zr samples unmodified and modified using laser radiation with the obtained shallow and deepest laser-modified layer are listed in Table 3. All results of contact angle measurements prove the hydrophilicity of the tested materials, which is necessary for materials intended for use in implantology and prosthetics in order to improve their osseointegration with bone tissue [73]. Analyzing the wettability tests, it is noted that the laser melting of the Ti13Nb13Zr alloy causes an increase in the contact angle of the material as in [52] to a value of about 80°. It can be also mentioned that the parameters affecting the deepening of the laser-melted layer causes a smaller increase in the contact angle. The carbon enrichment of the surface layer of the Ti13Nb13Zr alloy reduced the dynamics of the contact angle increase compared to laser melting. A carburized sample with the deepest laser-melted layer showed only a slight increase in the contact angle relative to the MR reference. Studies [74,75,76] assess that contact angles between 35° and 80° are beneficial for materials cooperating with bone tissue. The best contact angle values according to other publications are 40–60° [77] depending on cells and for bone cells at 35 ÷ 85°, with the optimum value at 55° [78]. Therefore, all tested samples demonstrating the contact angles between 46° and 82° possess hydrophilic surfaces suitable for adhesion of cells. The difference between T2, T4, C2 and C4, in their wettability can be attributed to the positive and joined effect of the significant laser remelting at high laser power and the presence of MWCNTs. Therefore, positive effects of laser carburizing of the Ti13Nb13Zr alloy in the perspective of using this alloy as a biomaterial are seen.

### 3.5. Nanomechanical Studies

Table 4 presents the average values of the tested properties, i.e., nanohardness and Young’s modulus (reduced and real) and the maximum penetration of the nanoindenter into the analyzed material for individual areas of the sample comprising the laser-melted layer, the layer affected by the heat of the laser beam and the material below that the laser did not affect. The values presented are accompanied by deviations consisting of the values of the largest and lowest recorded measurement.

As the nanohardness increases, approaching the sample face, smaller penetration depths of the indenter are observed, confirming the hardening of the material. Approaching the forehead, a decrease in the Young′s modulus value is also observed, which is beneficial because this value approaches the value of the Young′s modulus of bone tissue. Analyzing the nanohardness, an increase is observed as the laser pulse power increases. For laser melting with the shallowest laser-melted layer, an increase in nanohardness of about 25% was obtained and a further 25% with a more than double increase in laser pulse power, where the deepest laser-melted layer was observed compared to MR. In the case of laser carburizing for a sample with the shallowest laser-melted layer, an increase in nanohardness of more than 40% is observed compared to a laser-melted sample with the same parameters. By more than doubling the laser pulse power, another 10% increase in nanohardness is achieved, and as a result, almost 30% relative to laser melting with the same parameters. Therefore, it is noted that laser carburizing increases the value of nanohardness more significantly than laser melting. In the case of Young′s modulus, more desirable values were obtained with laser melting. With laser alloying, the decrease was small relative to the base material (material that was not affected by the laser in Table 4).

In [67,68], where the source of carbon was graphite, with higher laser pulse powers and more numerous titanium carbide structures observed, microhardness values of 4 GPa were obtained in zones with a direct penetration of up to 12 GPa in the layer at the penetration face. In [44], where a coating of carbon nanotubes from a solution with a higher percentage of MWCNTs was used, surface nanohardness values between 5 and almost 9 GPa were obtained using other laser parameters.

**Figure 9:**
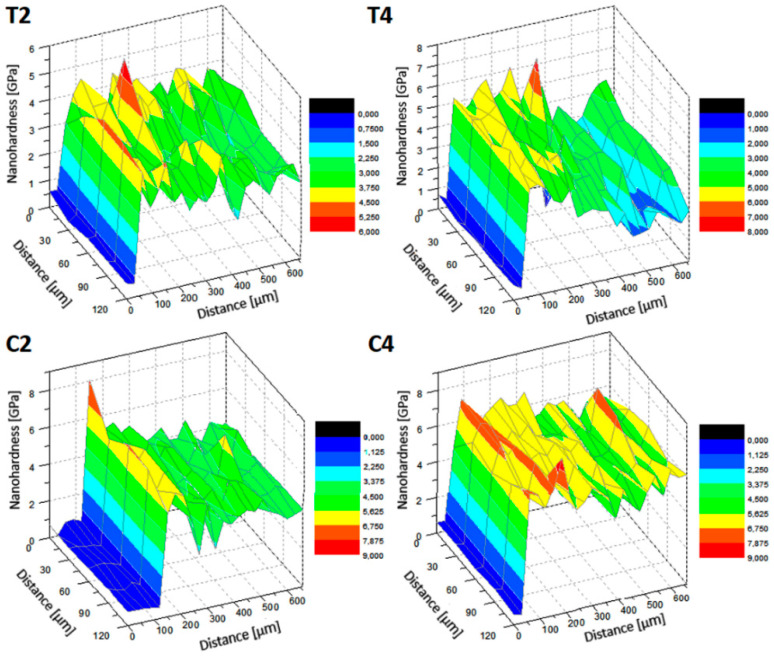
3D distribution of nanohardness for tested samples T2, T4, C2 and C4.

**Figure 10:**
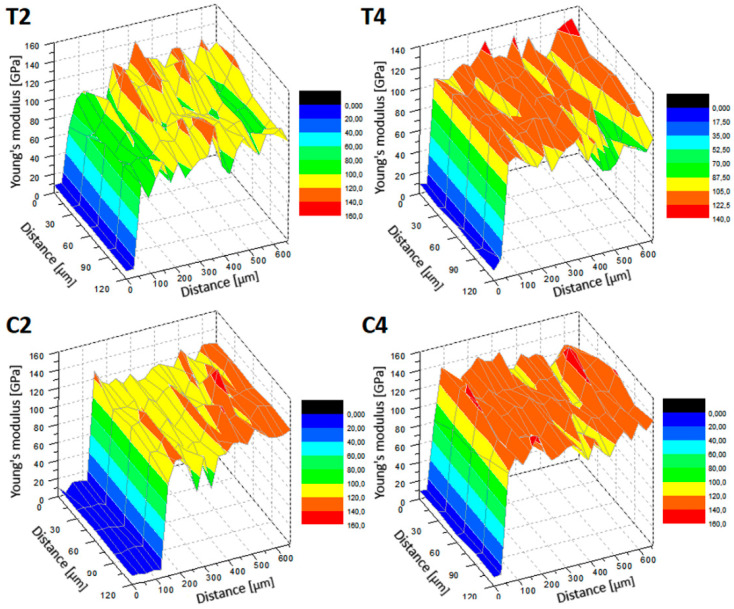
3D distribution of reduced Young’s modulus for tested samples T2, T4, C2 and C4.

The 3D distributions of nanohardness and Young′s modulus values recorded on the nanoindenter are shown in Figure 9 and Figure 10. Observing the 3D distribution of nanohardness, several initial significantly different measurements should be omitted, which are the result of penetration of the depth gauge into the resin. By analyzing the proper material, it can be confirmed that for each tested sample there was an increase in nanohardness in the laser-modified layer. The shallowest cure occurred for the C2 carburized sample. In samples T2 and T4, despite the difference in the depth of the modified layer and its tested nanohardness, it can be seen that the depth of the hardened layer is similar. The deepest hardened area is observed in the C4 alloyed sample (Figure 9). By analyzing 3D distributions of Young′s modulus values, disregarding the first 50 ÷ 100 μm which refers to the resin, it is confirmed that for each sample tested, the values of the elastic modulus decrease with the approach of the laser-melted front. Even in the T4 sample, for which the average values indicated a Young′s modulus higher in the modified zone than the area below it, it is noted that this property reaches higher values as it moves away from the sample front. Smaller Young′s modulus values occur in laser-modified layers without stopping with T2 and T4.

### 3.6. Corrosion Tests Analysis

**Figure 11:**
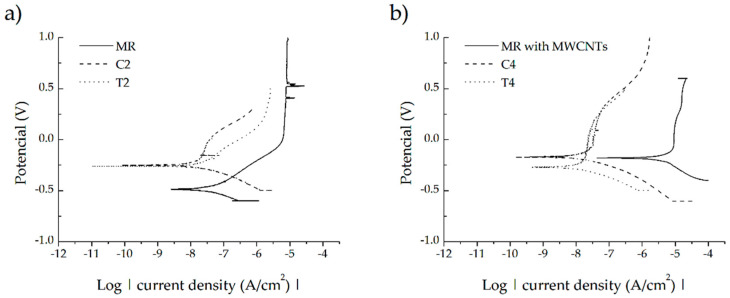
Potentiodynamic polarization curves in Ringer′s solution at room temperature of: (**a**) reference sample Ti13Zr13Nb, C2, T2 laser-modified layers; (**b**) reference sample Ti13Zr13Nb with MWCNTs coating, C4, T4 laser-modified layers.

Corrosion tests were carried out for uncoated and MWCNTs-coated Ti13Nb13Zr samples unmodified and modified using laser radiation with the shallowest and deepest laser-melted layers (T2, T4, C2, C4). Potentiodynamic curves for the tests performed are shown in Figure 11. Corrosion current density (I_corr_) and corrosion potential (E_corr_) values are shown in Table 5.

The improvement of corrosion resistance is demonstrated by a decrease in the value of the corrosion current and an increase in the value of the corrosion potential. An increase in the value of corrosion potential, but also an increase in the corrosion current was obtained for the examined alloy with deposited carbon coating. Thus, covering the alloy with a carbon coating does not guarantee an improvement in corrosion resistance. The increase in the value of the corrosion potential and the decrease in the value of the corrosive current is observed for samples subjected to laser treatment with and without a carbon coating. This demonstrates the improvement of the corrosion resistance of Ti13Nb13Zr due to laser processing. In the case of the analysis of the change in the value of corrosion potential, a certain relationship is noticed regarding the laser treatment of the material with and without a carbon coating. Higher values are obtained for laser-alloyed samples relative to laser melting with the same parameters. The highest and most desirable value was obtained for a carburized C4 sample with the deepest laser-melted layer. When analyzing the change in the value of corrosive current density, no clear relationship between laser alloying and laser melting is seen. The smallest, and therefore the most desirable value of corrosive current is observed for sample T4.

## 4. Conclusions

Using a method of electrophoretic deposition, a uniform coating of functionalized multi-walled carbon nanotubes was successfully deposited on the Ti13Nb13Zr alloy. By using laser melting methods, changes in the microstructure of this alloy, primarily grain refinement, were successfully achieved. Carburizing successfully enriched the surface layer of this alloy with carbon, which resulted in the formation of a new component—titanium carbide—whose forms depend on the processing parameters.

The laser processing parameters have a significant impact on the depth and shape of the laser-melted layer. An increase in the laser pulse power and its duration affects the deepening of the modified layer, while an increase in feed speed and a decrease in frequency causes it to become shallower. No cracks were observed in the laser-modified areas for any of the investigations. Changes in the microstructure in the laser-melted layer caused an increase in nanohardness of about 25% over the base material. Laser alloying caused an additional increase in nanohardness of about 25% compared to laser melting with the same process parameters. A reduction of the Young′s modulus value in laser-modified layers was achieved.

Corrosion tests showed that the applied laser treatment (laser remelting and laser alloying–carburizing) caused an increase in the value of corrosion potential and a decrease in the value of corrosive current density. This indicates better corrosion resistance, compared to the reference. Laser melting increased the contact angle to almost the hydrophilicity limit. For laser carburizing, a reduction in contact angle relative to laser melting was observed.

The results of the presented research lead to the conclusion that laser modification of the Ti13Nb13Zr alloy coated with carbon nanotubes may be an interesting alternative to the complicated and expensive techniques of producing coatings for applications in implants. Laser surface carburization promotes an increase in nanohardness, a decrease in Young′s modulus and obtaining a hydrophilic surface—the properties desired for the indicated application. The success of further research will depend on the results of the biologic research that is critical to the foreseen application.

## Figures and Tables

**Table 1:** Chemical composition of Ti13Nb13Zr titanium alloy in % by weight.

Chemical Element	C	Fe	N	O	Zr	Nb	Ti
Alloy	Chemical Composition (% wt.)						
Ti13Nb13Zr	0.04	0.05	0.019	0.11	13.0	13.0	72.78

**Table 2:** Parameters of laser processing of the tested material.

Sample	Material	Power of the Laser Pulse (W)	Duration of the Laser Pulse (ms)	Feed Speed (mm/s)	Frequency (Hz)
T1, C1	T-Ti13Nb13Zr alloy, C-Ti13Nb13Zr alloy with MWCNTs coating	800	2.25	3.00	25
T2, C2		700	3.25	6.00	25
T3, C3		700	4.00	3.00	25
T4, C4		2000	2.25	3.00	25
T5, C5		1000	3.25	6.00	25
T6, C6		2000	2.25	3.00	10

**Table 3:** Average contact angle values for the tested samples.

Sample	Average Contact Angle (°)
MR	47.1 ± 1.1
T2	81.6 ± 0.9
T4	77.0 ± 0.8
C2	77.0 ± 1.1
C4	46.1 ± 3.6

**Table 4:** Mechanical and nanoindentation properties of the tested samples.

Area	Sample	Nanohardness (GPa)		Reduced Young’s Modulus, Er (GPa)		Real Young’s Modulus, E (GPa)		Maximum Depth of Indentation (nm)	
Laser-melted layer	T2	4.08	+0.72 −0.91	90.61	+10.58 −18.86	85.72	+10.88 −19.10	796.00	+31.12 −52.95
	T4	4.98	+1.03 −1.65	111.05	+13.52 −17.22	107.07	+14.57 −17.22	722.55	+154.92 −164.58
	C2	5.83	+2.25 −1.96	115.11	+13.23 −16.43	111.42	+14.37 −17.44	678.90	+138.87 −97.02
	C4	6.36	+2.37 −1.78	126.12	+25.09 −29.67	123.51	+28.09 −42.12	647.94	+128.02 −81.38
Layer affected by the heat of the laser beam	T2	3.92	+0.82 −0.57	104.96	+13.74 −9.14	100.62	+14.64 −9.61	812.05	+57.46 −78.07
	T4	3.79	+2.34 −1.46	116.21	+41.58 −26.49	112.75	+46.50 −28.03	816.70	+216.97 −237.58
	C2	4.51	+1.13 −1.53	109.33	+14.80 −20.16	105.28	+15.87 −21.13	765.10	+159.98 −87.53
	C4	5.80	+0.95 −1.20	127.76	+11.83 −13.66	125.19	+13.15 −14.91	667.52	+79.28 −48.62
Material that was not affected by the laser	T2	3.27	+1.26 −1.30	113.70	+29.82 −25.35	110.02	+32.77 −26.99	879.79	+238.07 −226.77
	T4	2.92	+1.57 −1.22	105.83	+26.35 −25.10	101.70	+28.35 −26.10	947.87	+259.87 −204.44
	C2	4.31	+2.02 −1.10	124.80	+29.48 −15.21	121.97	+33.20 −16.50	765.42	+111.24 −137.40
	C4	5.70	+1.86 −1.17	128.83	+23.80 −17.39	126.41	+28.84 −18.97	674.64	+79.26 −91.28

**Table 5:** Corrosion current density and corrosion potential of T2, T4, C2 and C4 laser-modified layers, non-laser-modified Ti13Zr13Nb substrate (MR) and MWCNTs coating.

Sample	Corrosion Parameters	
	E_corr_ (V)	I_corr_ (A/cm^2^)
MR	−4.87 × 10^−1^	5.19 × 10^−8^
MR with MWCNTs coating	−1.80 × 10^−1^	3.52 × 10^−6^
T2	−2.55 × 10^−1^	3.47 × 10^−8^
T4	−2.69 × 10^−1^	2.33 × 10^−8^
C2	−2.45 × 10^−1^	3.10 × 10^−8^
C4	−1.63 × 10^−1^	4.22 × 10^−8^
